# Diagnostic accuracy of periapical radiography and panoramic radiography in the detection of apical periodontitis: a systematic review and meta-analysis

**DOI:** 10.1007/s11547-024-01882-z

**Published:** 2024-09-03

**Authors:** Giulio Stera, Martina Giusti, Andrea Magnini, Linda Calistri, Rossana Izzetti, Cosimo Nardi

**Affiliations:** 1 Florence, Italy; 2 Department of Experimental and Clinical Medicine, University of Florence, 50134 Florence, Italy; 3 Radiodiagnostic Unit n. 2, Department of Experimental and Clinical Biomedical Sciences, University of Florence-Azienda Ospedaliero-Universitaria Careggi, Largo Brambilla 3, 50134 Florence, Italy; 4 Unit of Dentistry and Oral Surgery, Department of Surgical, Medical and Molecular Pathology and Critical Care Medicine, University of Pisa, 56126 Pisa, Italy

**Keywords:** Apical periodontitis, Two-dimensional imaging, Periapical radiography, Panoramic radiography, Diagnostic accuracy

## Abstract

**Objective:**

Apical periodontitis (AP) is one of the most common pathologies of the oral cavity. An early and accurate diagnosis of AP lesions is crucial for proper management and planning of endodontic treatments. This study investigated the diagnostic accuracy of periapical radiography (PR) and panoramic radiography (PAN) in the detection of clinically/surgically/histopathologically confirmed AP lesions.

**Method:**

A systematic literature review was conducted in accordance with the PRISMA guidelines. The search strategy was limited to English language articles via PubMed, Embase and Web of Science databases up to June 30, 2023. Such articles provided diagnostic accuracy values of PR and/or PAN in the detection of AP lesions or alternatively data needed to calculate them.

**Results:**

Twelve studies met inclusion criteria and were considered for the analysis. The average value of diagnostic accuracy in assessing AP lesions was 71% for PR and 66% for PAN. According to different accuracy for specific anatomical areas, it is recommended to use PR in the analysis of AP lesions located in the upper arch and lower incisor area, whereas lower premolar and molar areas may be investigated with the same accuracy with PR or PAN.

**Conclusions:**

Two-dimensional imaging must be considered the first-level examination for the diagnosis of AP lesions. PR had an overall slightly higher diagnostic accuracy than PAN. Evidence from this review provided a useful tool to support radiologists and dentists in their decision-making when inflammatory periapical bone lesions are suspected to achieve the best clinical outcome for patients, improving the quality of clinical practice.

## Introduction

Apical periodontitis (AP) is one of the most common pathologies of the oral cavity [1]. AP is a local inflammatory lesion in the periapical area due to bacterial infections of the root canal system [2, 3]. The main causes of AP lesions are deep dental caries, root fractures and endodontic treatments [4, 5]. AP lesions can be both asymptomatic and symptomatic, or rather accompanied by pain, swelling and tenderness in affected teeth [6, 7]. If AP lesions are not treated properly, they can lead to serious dental health issue complications such as apical abscesses and tooth loss [8, 9]. An early and accurate diagnosis of AP lesions is crucial for proper management and planning of endodontic treatments to prevent such complications [10–12]. Biopsy is considered the gold standard examination to diagnose AP lesions, but it is an invasive procedure characterized by risks and complications [13, 14]. Inflammation and immune reactions in the periapical tissue cause resorption of the surrounding bone detected as a radiolucent area on X-ray imaging [15]. Several radiological diagnostic techniques have been developed to identify AP lesions [16], including both two-dimensional techniques represented by periapical radiography (PR) and panoramic radiography (PAN) [17, 18].

PR is an intraoral imaging technique in which films or sensors are placed inside patients’ mouth close to the teeth and surrounding bone, allowing for detailed visualization of individual teeth and their apices. This technique is particularly useful to diagnose periapical bone disease, assess root canal anatomy and detect root fractures or bone loss [19]. Its higher spatial resolution than PAN makes PR ideal for detailed evaluations, but the very small field of view that characterizes it limits its use to small anatomical areas*.* Therefore, PR favors its use for the identification of periapical abnormalities, although it is not able to offer a complete view of dental arches [20].

In contrast, PAN is an extraoral imaging technique in which X-ray tube rotates around patients’ head capturing a broad view of the entire dental arches and surrounding structures on a single film. In fact, PAN provides a complete view of dental arches allowing the evaluation of tooth structures, periapical tissues and bone jaws [21]. This wide coverage is advantageous for comprehensive assessments of dental development, trauma and alterations affecting jaws or multiple teeth such as cysts, tumors and impacted teeth. However, PAN has lower spatial resolution than PR making it less effective for the detection of fine details like early carious lesions or small changes in bone structure [22]. In addition, PAN has some limitations in terms of accuracy in the identification of periapical abnormalities because of the overlap of soft tissues and bone structures due to both individual morphological conditions [23, 24] and patients’ positing problems [25, 26].

The introduction of cone beam computed tomography (CBCT) as three-dimensional imaging technique enables a detailed volumetric visualization of teeth and surrounding bone structures [27–29] allowing an excellent identification of AP lesions [30]. Nevertheless, the higher purchase costs and the higher radiation dose delivered by CBCT than two-dimensional techniques make CBCT an effective second-level examination recommended in individual cases [31]. In clinical practice, radiologists and dentists have to know how accurate are PR and PAN in the identification of AP lesions in relation to their better cost-effectiveness and low-dose levels than CBCT [32–34].

This systematic review aimed to investigate the diagnostic accuracy of two-dimensional imaging techniques, namely PR and PAN, in the detection of AP lesions. The secondary objective was to provide a useful tool to support radiologists and dentists in their decision-making when inflammatory periapical bone lesions are suspected in order to achieve the best clinical outcome for patients.

## Materials and methods

### PIRO question and literature searches

This systematic literature review was conducted in accordance both with the PRISMA guidelines (Preferred Reporting Items for Systematic reviews and Meta-Analyses) and a pre-specified protocol registered on the PROSPERO database [35].

The PRISMA statement consists of a 27-item checklist and a four-phase flow diagram. The checklist includes items deemed essential for transparent reporting of a systematic review. The registration number in PROSPERO database was CRD42023395948. PIRO strategy is commonly applied for the development of an adequate research question and bibliographic research according to the scope of synthesis review [36, 37].

The question that we set was as follows.

(P) Are periapical radiography and panoramic radiography.

(I) adequate for the detection of confirmed AP lesions.

(R) using different reference standards (CBCT examinations, histopathology, neural networks).

(O) to assess diagnostic accuracy of such two-dimensional imaging techniques?

The search strategy was conducted up to June 30, 2023. It was limited to English language articles via PubMed, Embase and Web of Science databases. Crucial terms to understand the current review were defined as follows:

- Periapical radiography. An imaging technique that uses focused X-rays to obtain a detailed image of the tooth root and surrounding bone tissues.
- Panoramic radiography. An imaging technique that provides a complete view of the entire dental arches and bone jaws in a single radiogram.
- Apical periodontitis. Local inflammation of the bone tissue surrounding the apical third of the root.
- Diagnostic accuracy. The ability of a test or investigation to provide correct and reliable results in the process of diagnosing a specific condition or pathology.

The electronic search was carried out using a series of keywords including Periapical Radiograph*, Intraoral Radiograph*, Panoramic Radiograph*, Orthopantomograph*, OPT, Apical Periodontitis and Periapical Periodontitis.

Search terms were combined using the Boolean operator "OR" to group terms related to different imaging techniques (“Periapical Radiograph*,” “Intraoral Radiograph*,” “Panoramic Radiograph*,” and “Orthopantomograph*”). Additionally, the Boolean operator "OR" was used for terms indicating AP lesions ("Apical Periodontitis" and "Periapical Periodontitis"). Subsequently, results of these two searches were combined using the Boolean operator "AND" to obtain a cross-referenced search between imaging techniques and AP lesions. A detailed explanation of the search strategies is given in Table 1.

**Table 1:** Search strategy

Indexing terms	Publications (N)		
Search string	PubMed	Embase	Web of Science
#01 Periapical radiograph*	307	272	373
#02 Intraoral radiograph*	312	325	320
#03 Panoramic radiograph*	1,564	1,551	1,399
#04 Orthopantomograph*	317	346	85
#05 OPT	842	1,179	2,851
#06 Apical periodontitis	1,008	991	838
#07 Periapical periodontitis	80	94	88
#08 = #01 OR #02 OR #03 OR #04 OR #05	3,322	3,655	4,944
#09 = #06 OR #07	1,088	1,085	900
#10 = #08 AND #09	14	12	21

## Inclusion and exclusion criteria

We collected studies published in international peer-reviewed journals that included at least one of PR and PAN and provided diagnostic accuracy of such imaging techniques in the detection of AP lesions. In cases of diagnostic accuracy values were not directly reported, data used to calculate them were alternatively used. To be analyzed in our systematic review, studies had to meet specific inclusion and exclusion criteria reported in Table 2.

**Table 2:** Adopted criteria to select articles for systematic review

Inclusion criteria	Exclusion criteria
• Patients or cadaver tissues	• Samples made up of animals
• PR and/or PAN as diagnostic tools to detect AP lesions	• Periapical radiolucency not identified as AP lesions by clinical, surgical or histopathological examinations
• Diagnostic accuracy values for the detection of AP lesions or data used to calculate them	• AP lesions not identified via PR and PAN
• Clinical, surgical or histopathological confirmation of AP lesions	• AP lesions previously diagnosed for other reasons
	• No diagnostic accuracy values for the detection of AP lesions or data used to calculate them
	• Articles not written in English language
	• Reviews, short communications, letters to the editor, case reports

## Study selection and data extraction

Two readers (G.S. and M.G.) independently examined titles and abstracts to determine their eligibility for inclusion. Manual search was conducted by them using the references of the articles resulting from the database searches. Duplicates were removed through a manual cross-analysis. Twenty-seven articles were selected for evaluation. Screening the full text was done whenever the abstract did not give enough information to define eligibility. Moreover, the full text was read when at least one of the authors claimed that the study met the inclusion criteria. A third reviewer independently checked and evaluated the decisions on studies inclusion (C.N.). In case of disagreement on study selection or data extraction, all reviewers discussed together the issue and reach consensus.

Data were individually extracted from each study on: (1) study author and year of publication; (2) diagnostic confirmation of AP lesions including clinic, surgery or histopathology; (3) reference standard for the assessment of diagnostic accuracy values; (4) bi-dimensional imaging technique under investigation used to assess the diagnostic accuracy; (5) equipment model used for imaging; (6) sample size—total number of analyzed teeth; (7) the presence of treated or non-treated teeth with endodontic therapy; (8) in vivo or ex vivo models; (9) scores used to assess AP lesions; and (10) size of AP lesions. No software systems or tools were used for data extraction and management. Diagnostic accuracy values of the imaging techniques were reported according to the available data in each study (Table 3). Diagnostic accuracy values were transcribed when they were directly presented in the selected articles. Instead, diagnostic accuracy was calculated when its value was missing, but data for its measurement were available. The process of the diagnostic accuracy calculation for the detection of AP lesions for each study included in the review is explained in detail in Appendix 1. Similarly, sensitivity, specificity, positive and negative predictive values were calculated when missing.

**Table 3:** Data extracted from the articles included in the current review

Author and year of publication	Diagnostic confirmation of AP	Reference to assess accuracy	Imaging technique under investigation	Equipment model	Sample size	Treated or Untreated teeth	In vivo or ex vivo	AP score	AP size
Estrela et al, 2008 [50]	Clinic	CBCT	PR, PAN	Max S-1 X-ray equipment, Veraviewepocs panoramic X-ray unit	1508	Both	In vivo	PAI	No
Estrela et al, 2009 [43]	Clinic	CBCT	PR	Spectro x70 2X-ray unit	1020	Treated	In vivo	NA	No
Moura et al, 2009 [44]	Clinic	CBCT	PR	NA	300	Treated	In vivo	NA	No
Weissman et al, 2015[45]	Clinic	CBCT	PR	Kodad 6100 (Carestream)	67	Untreated	In vivo	NA	No
Kanagasingam et al, 2017 [39]	Histophatology	Histophatology	PR	Heliodent DS Intraoral X-ray system	67	NA	Ex vivo	NA	yes
Kanagasingam et al, 2017 [40]	Histophatology	Histophatology	PR	Heliodent DS Intraoral X-ray system	67	NA	Ex vivo	NA	yes
Nardi et al, 2017 [47]	Clinic	CBCT	PAN	Orthoceph OC200 D	240	Untreated	In vivo	PAI	Yes
Nardi et al, 2018 [48]	Clinic/Surgery	CBCT	PAN	Orthoceph OC200 D	480	Treated	In vivo	PAI	Yes
Nardi et al, 2020 [49]	Clinic/Surgery	CBCT	PAN	Orthoceph OC200 D	480	Both	In vivo	PAI	Yes
Jang et al, 2020 [46]	Clinic	CBCT	PR	Kodad 6100 (Carestream)	203	Untreated	In vivo	PAI	Yes
Bosoni et al, 2021 [42]	Clinic/Surgery	MSCT	PAN	Orthoceph OC200 D	644	NA	In vivo	NA	No
Li et al, 2022 [41]	Clinic	PR	PR*	NA	419	Untreated	In vivo	NA	No

CBCT: Cone beam computed tomography. MSCT: Multislice computed tomography. PR: Periapical radiography. PAN: Panoramic radiography. AP: Apical periodontitis. PAI: periapical index. NA: Not achievable since no reference standard is described.

*PR exploiting artificial intelligence in the identification of AP lesions

## Statistics

The decision to perform meta-analyses was made depending on the availability of data on diagnostic accuracy of PAN and PR. In the meta-analysis, a random-effects model was used to calculate a pooled diagnostic accuracy for both groups with a 95% confidence interval. The inconsistency index I-square (*I*^2^) test was used to assess heterogeneity of diagnostic accuracy for each study. OpenMeta [Analyst] (http://www.cebm.brown.edu/open_meta/open_meta/open_meta) software was employed to perform meta-analyses. P-values < 0.05 were considered statistically significant. Graphical illustration and summary of meta-analyses were provided through forest plots.

## Risk of bias

To assess the risk of bias we applied a quality assessment tool called QUADAS-2 (Quality Assessment Tool for Diagnostic Accuracy Studies-2), a system specifically developed for systematic reviews of accuracy studies [38]. Two reviewers (L.C. and A.M.) independently assessed the risk of bias for each of the articles included in the review. Discrepancies between the reviewers' assessments were resolved by involving a third independent reviewer (C.N.).

The use of the QUADAS-2 method offered a complete and effective framework for the assessment of the bias’s potential risk of the studies included in the current systematic review, ensuring reliability of the conclusions reached.

## Results

### Study selection

**Fig. 1:**
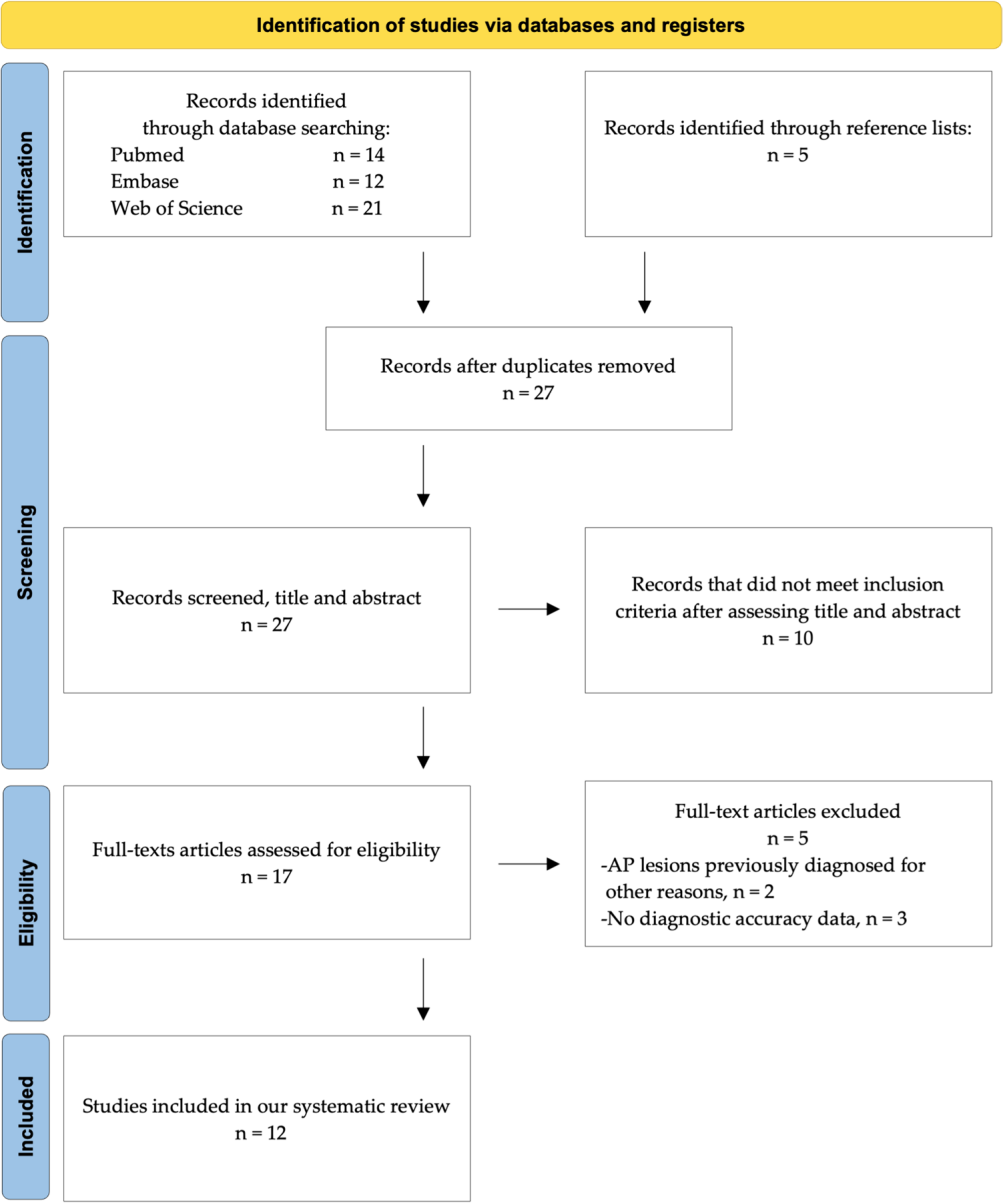
Flowchart consistent with preferred reporting items for systematic review (PRISMA) statement

Fourteen, twelve and twenty-one studies were identified from PubMed, Embase and Web of Science, respectively (Fig. 1). Five articles were also found from manual search using the bibliographic references of the articles resulting from the database searches. Duplicates were removed.

Twenty-seven articles were selected for evaluation. Ten studies were excluded for not meeting the inclusion criteria based on titles and abstracts. Of the remaining seventeen studies, the reviewers examined the full text. Five articles were further excluded since two of them had AP lesions previously diagnosed for other reasons, while the other three did not have diagnostic accuracy data.

Finally, twelve studies met our inclusion criteria and were included for the final analysis. Such studies were published between 2008 and 2022.

## Data extraction

Two studies used histopathology as diagnostic method of AP lesions in cadaver tissues [39, 40], whereas all the other studies performed clinical and/or surgical assessments in being humans. Li and colleagues [41] developed a tool of artificial intelligence for the assessment of AP lesions on PR.

About reference standard, studies on cadavers employed histopathology [39, 40]. Bosoni et al. [42] used multislice spiral computed tomography (MSCT), while Li et al. [41] used PR exploiting artificial intelligence in the identification of AP lesions. All the other studies adopted CBCT as the reference standard to detect AP lesions.

As regards imaging techniques, seven [39–41, 43–46] and four [42, 47–49] studies used PR and PAN to detect AP lesions, respectively. One study used both PR and PAN [50]. Furthermore, three studies involved teeth previously subjected to endodontic treatment [43, 44, 48], whereas four studies considered teeth with no endodontic treatment [41, 45–47]. Two studies included both treated and untreated teeth [49, 50]. Such information was not provided in three studies [39, 40, 42].

Only five studies reported a specific score called periapical index (PAI) for the identification of AP lesions [46–50]. PAI assessed the condition of periapices by assigning a value from 1 (normal) to 5 (severe periodontitis with aggravating features) based on the presence or the absence of specific radiographic features [51]. PAI scores between 2 and 5 were considered indicative of AP lesions. In the studies by Nardi et al. [47–49], PAI 2 and 3 scores were pooled together as well as PAI 4 and 5 scores in order to simplify their analysis.

Finally, six studies provided information on the size of AP lesions [39, 40, 46–49].

In the two studies by Kanagasingam et al. [39, 40], which referred to the same group of examined teeth, the average diameter of AP lesions was 2.7 mm. In these studies, most AP lesions were considered “small” as 60% of them were smaller than 2.5 mm in diameter. In the three articles by Nardi et al. [47–49], AP lesions were divided into small (2–4.5 mm) and large (4.6–7 mm) bone alterations. Jang et al. [46] made a distinction between lesions with a diameter smaller and larger than 2 mm (Table 3).

## Diagnostic accuracy

In eight and five studies analyzed in the systematic review, it was possible to have information on diagnostic accuracy of PR [39–41, 43–46, 50] and PAN [42, 47–50], respectively. In ten of them, sensitivity, specificity, positive and negative predictive values were reported or data to calculate them were available. Estrela et al. [50] were the only that assessed diagnostic accuracy of both PR and PAN.

In Table 4, both found and calculated diagnostic accuracy, sensitivity, specificity, positive and negative predictive values were described (see also Appendix 1 for detailed calculation processes).

**Table 4:** Diagnostic accuracy, sensitivity, positive predictive value and negative predictive value of periapical radiography (PR) and panoramic radiography (PAN)

Author and year of publication	Diagnostic accuracy	Sensitivity	Specificity	Positive predictive value	Negative predictive value
Estrela et al., 2008 [50]	70.0% for PR 54.0% for PAN	55.0% for PR 28.0% for PAN	98.0% for PR 100% for PAN	98.0% for PR 99.0% for PAN	55.0% for PR 44.0% for PAN
Estrela et al., 2009 [43]	64.7% for PR*	–	–	–	–
Moura et al., 2009 [44]	74.3% for PR*	–	–	–	–
Weissman et al., 2015[45]	71.7% for PR*	72.0%*	100%*	100%*	48.0%*
Kanagasingam et al., 2017 [39]	64.1% for PR*	29.5%*	99.5%*	99.5%*	40.5%*
Kanagasingam et al., 2017 [40]	65.9% for PR*	32.5%*	99.0%*	99.0%*	41.5%*
Nardi et al., 2017 [47]	65.0% for PAN	34.2%	95.8%	89.1%	59.3%
Nardi et al., 2018 [48]	71.3% for PAN	48.8%	93.8%	88.6%	64.7%
Nardi et al., 2020 [49]	70.0% for PAN*	45.9%*	96.3%*	92.4%*	64.2%*
Jang et al., 2020 [46]	69.4% for PR*	–	–	–	–
Bosoni et al., 2021 [42]	72.1% for PAN	46.8%	97.4%	94.7%	64.7%
Li et al., 2022 [41]	83.0% for PR*	82.0%	84.0%	83.7%	82.4%

^*^Calculated by the current researchers based on the data collected from the included studies

**Fig. 2:**
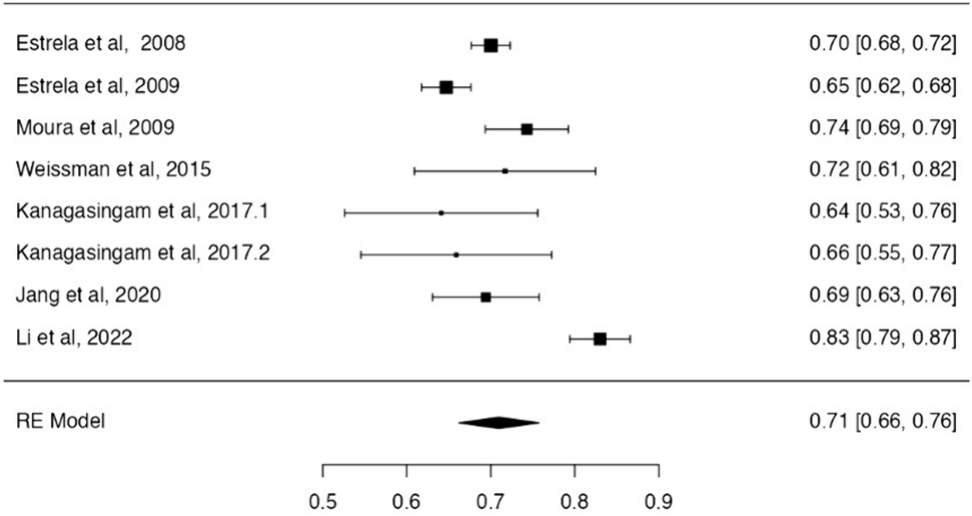
Forest plot from random effects of meta-analysis evaluating the diagnostic accuracy of PR (95% confidence interval [CI])

**Fig. 3:**
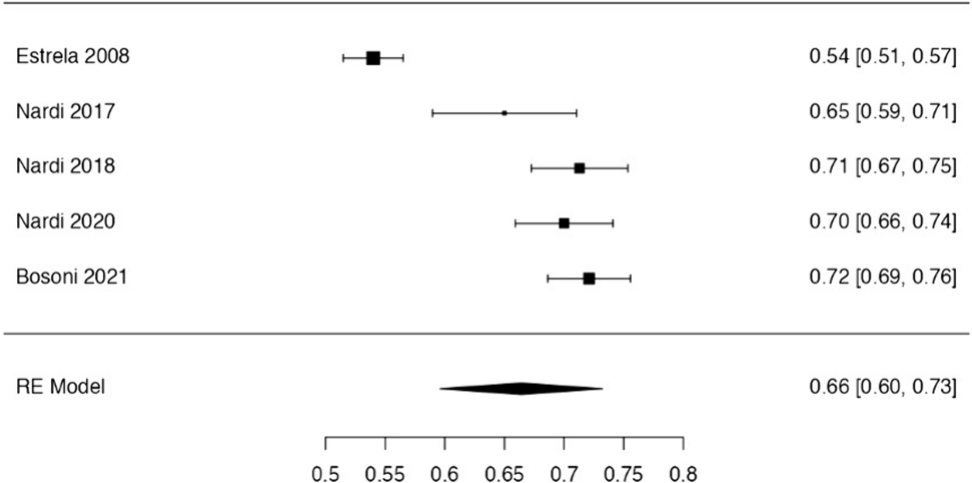
Forest plot from random effects of meta-analysis evaluating the diagnostic accuracy of PAN (95% confidence interval [CI])

Two separate meta-analyses were performed for studies assessing the diagnostic accuracy of PR (*n* = 8 studies [39–41, 43–46, 50]) and PAN (*n* = 5 studies [42, 47–50]), respectively. The diagnostic accuracy of PR and PAN was 71% (CI 95%: [66%–76%], *p* < 0.01, *I*^2^ = 87.2%) (Fig. 2) and 66% (CI 95%: [60%–73%], p < 0.01, *I*^2^ = 93.92%) (Fig. 3).

Three studies reported diagnostic accuracy for anatomical site, namely incisor, canine, premolar and molar areas [47, 48, 50], while diagnostic accuracy for specific anatomical sites was calculated in other two studies [44, 49]_._

Diagnostic accuracy was measured for anatomical sites as incisor, canine, premolar and molar areas [44, 47–50]. PR diagnostic accuracy was generally higher than PAN one, especially in incisors. In the three studies by Nardi et al. [47–49], PAN diagnostic accuracy was also calculated distinguishing between upper (average diagnostic accuracy 63.1%) and lower (average diagnostic accuracy 75.1%) jaws. Such parameters are given in Table 5.

**Table 5:** Diagnostic accuracy of periapical radiography (PR) and panoramic radiography (PAN), for anatomical areas

Author and year of publication	Incisors	Canines	Premolars	Molars
Estrela et al., 2008 [50]	67.0% for PR 42.0% for PAN	75.0% for PR 61.0% for PAN	74.0% for PR 59.0% for PAN	67.0% for PR 51.0% for PAN
Estrela et al., 2009 [43]	–	–	–	–
Moura et al., 2009 [44]	67.3% for PR *	67.3% for PR *	80.3% for PR *	75.2% for PR *
Weissman et al., 2015[45]	–	–	–	–
Kanagasingam et al., 2017 [39]	–	–	–	–
Kanagasingam et al., 2017 [40]	–	–	–	–
Nardi et al., 2017 [47]	55.0% upper arch for PAN 50.0% lower arch for PAN	67.5% upper arch for PAN 80.0% lower arch for PAN	67.5% upper arch for PAN 80.0% lower arch for PAN	57.5% upper arch for PAN 80.0% lower arch for PAN
Nardi et al., 2018 [48]	62.5% upper arch for PAN 60.0% lower arch for PAN	71.3% upper arch for PAN 85.0% lower arch for PAN	71.3% upper arch for PAN 85.0% lower arch for PAN	63.8% upper arch for PAN 85.0% lower arch for PAN
Nardi et al., 2020 [49]	61.3% upper arch for PAN* 70.0% lower arch for PAN *	71.3% upper arch for PAN * 85.0% lower arch for PAN *	71.3% upper arch for PAN * 85.0% lower arch for PAN	57.5% upper arch for PAN * 81.3% lower arch for PAN *
Jang et al., 2020 [46]	–	–	–	–
Bosoni et al., 2021 [42]	–	–	–	–
Li et al., 2022 [41]	–	–	–	–

^*^Calculated by the current researchers based on the data collected from the included studies

## Risk of bias

An overall medium/low risk of bias was found based on the parameters of the QUADAS-2 tool.

Most of the results with high risk of bias were found in the applicability column of the diagnostic study under consideration (“index test” column regarding applicability) [45].

This was because most of the studies did not have as primary objective the provision of data on the diagnostic accuracy of two-dimensional imaging techniques in the detection of AP lesions.

Most of the results with unclear risk of bias were found in the “flow and timing” column, which pertained to the pathway of patients analyzed [43, 45, 46, 49, 50]. The reasons of such risk of bias were especially associated with the lack description of the applied diagnostic protocols in the included studies into the systematic review. The paper by Moura et al. [44] was identified as being at high risk of bias. This was mainly attributable to the lack of clarity regarding the patient flow in the study (Table 6).

**Table 6:** Risk of bias, evaluation of quality related to included studies (QUADAS-2)

Author and year of publication	Risk of bias				Applicability concerns		
	Patient selection	Index test	Reference standard	Flow and timing	Patient selection	Index test	Reference standard
Estrela et al., 2008 [50]	V	V	V	?	V	V	V
Estrela et al., 2009 [43]	V	V	V	?	V	V	V
Moura et al., 2009 [44]	V	V	?	X	V	X	V
Weissman et al., 2015 [45]	V	?	V	?	V	X	?
Kanagasingam et al., 2017 [39]	?	V	V	V	?	X	V
Kanagasingam et al., 2017 [40]	?	V	V	V	?	X	V
Nardi et al., 2017 [47]	V	V	V	V	V	V	V
Nardi et al., 2018 [48]	V	V	V	V	V	V	V
Nardi et al., 2020 [49]	V	V	V	?	V	V	V
Jang et al., 2020 [46]	V	V	V	V	V	?	V
Bosoni et al., 2021 [42]	V	V	V	V	V	X	V
Li et al., 2022 [41]	V	V	?	V	V	X	X

*V Low Risk; X High Risk; ? Unclear Risk*

## Discussion

PR showed higher diagnostic accuracy than PAN—around 4%—in the diagnosis of AP lesions. This was intrinsically due to the different technical features of the two two-dimensional devices, especially the mechanism of imaging acquisition that influenced the spatial resolution [17, 21, 32]. PR offered greater spatial resolution and more concentrated focus on periapical area than PAN allowing for better visualization of AP lesions.

PR produced high-resolution images of periapical region around dental roots. Consequently, anatomical details and bone abnormalities as AP lesions were visualized more accurately on PR [27, 28, 34]. On the other hand, PAN had lower spatial resolution covering the whole mouth [23–25].

**Fig. 4:**
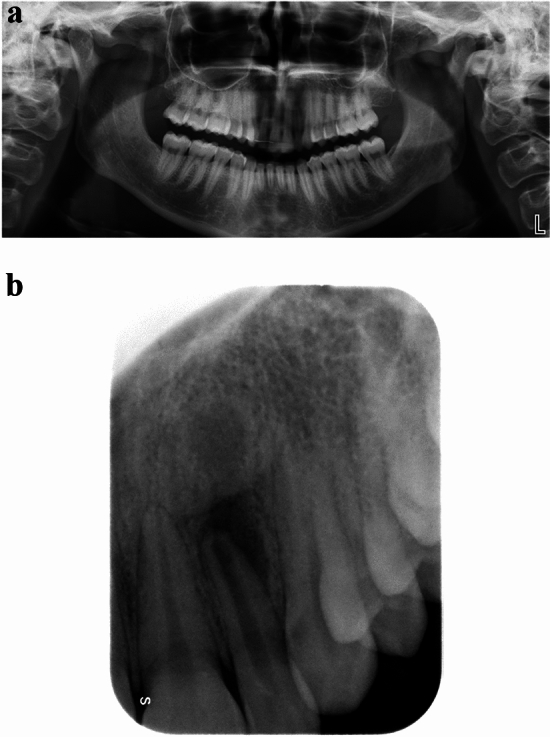
Apical periodontitis affecting the upper left lateral incisor. **A** In panoramic radiography, no periapical bone lesion was detected at the level of the periapex. A large area of radiolucency around incisors of both sides and especially around the root of both lateral incisors can be observed because of the overlap of the air inside the nasal cavity. **B** Same patient. In periapical radiography, changes in bone structure with clear mineral loss can be undoubtedly noticeable at the level of the periapex

Large differences in the detection of AP lesions depended on anatomical areas. While PAN had low diagnostic accuracy in the upper and lower incisor areas (42.0%–70.0%) and in the upper molar area (57.5%–63.8%), PR showed higher diagnostic accuracy in such anatomical sites (67.0%–75.2%) (Fig. 4). As far as the molar areas of lower jaws were concerned, both imaging techniques allowed good visualization of AP lesions thanks to the lower presence of superstructures, or rather the overlapping with other anatomical regions (i.e., air, spine, skull base, hyoid bone, nasal bone/cartilage and hard palate) [23–25]. The lower canine, premolar and molar areas were the areas where AP lesions were most easily identified (80.0%–85.0%). This was even more significant for PAN since its mechanisms of image formation are based on a curved rotational thick-layer tomography. A specific and punctual evaluation for each anatomical area, in fact, was crucial to obtain an accurate diagnosis of AP lesions.

Focusing on diagnostic accuracy of PAN for upper and lower arches, the lower canine and premolar areas had higher accuracy (mean value 83.3%) than the upper ones (mean value 70.0%). At the same time, regarding the evaluation of the size of AP lesions, no significant difference was found between the two analyzed types of imaging techniques. However, PAN showed lower capability in detecting small (2.0–4.5 mm) (mean value 72.4%) than large (4.6–7.0 mm) (mean value 81.6%) AP lesions because larger lesions resulted in more bone resorption, which appeared radiographically as an area of ​​radiolucency better perceivable within the radiopacity of bone tissue. This indicated that the size of bone resorption was a key element in detecting AP lesions by two-dimensional imaging.

It was more difficult a detailed visualization of AP lesions on PAN, especially when they had small size and were located in anterior areas of the mouth, such as the upper and lower incisors. Instead, AP lesions were more clearly visualized in the molar area on PAN because of the aforementioned limited overlapping of anatomical structures. In fact, a significant difference was observed between PR and PAN based on the different anatomical areas.

PR and PAN had similar diagnostic accuracy rates for endodontically treated and untreated teeth with slightly better results for PAN (PR mean diagnostic accuracy 69.9% for treated and 70.6% for untreated teeth; and PAN mean diagnostic accuracy 71.3% for treated and 65.0% for untreated teeth). This suggested that the presence of prior endodontic treatment influenced the visualization and detection of AP lesions. However, the few available data on this topic reduced the consistency of information.

In the study by Nardi et al. [49], endodontic treatments positively influenced the increase in true positives in untreated (67.1%) and treated (75.0%) teeth. This was attributable to two main factors. First, root fillings provided tracing of the pulp canal to the apex, allowing for delineation of the complete morphology of the root and apical periodontium, especially when the roots were curved or overlapping each other. Second, the use of endodontic therapies stimulated the formation of reactive condensing osteitis around AP lesions by enhancing the radiographic contrast between the radiolucency of the periapical bone lesion and the surrounding alveolar bone.

It was also important to underline the difference in diagnostic accuracy in the two studies by Kanagasingam et al. performed on the same tooth sample [39, 40]. The disparity of results in term of diagnostic accuracy of PR (64.1% and 65.9%) was due to the exclusive digital viewing of images in the second study [40], whereas in the first one images were both analogical and digital [39]. This difference in visualization modes explained the higher accuracy found in [40] since the use of digital images leaded to better image quality, possibilities of post-processing, reduced radiation exposure, greater efficiency and the ability to use of artificial intelligence for a more precise diagnosis. These observations on the evaluation of the studies included in the systematic review highlighted the need to carefully consider the different methodologies and variables influencing the diagnostic accuracy of two-dimensional radiographs in the detection of AP lesions.

A special attention must be reserved to the discussion of the contribution offered by Li et al. [41] that reported the diagnosis of AP lesions carried out by an artificial intelligence tool implemented on PR. The reference standard used to assess diagnostic accuracy was not histopathology or CBCT, but PR itself with clinically or surgically confirmed diagnosis.

PR and PAN currently remain the first-line imaging techniques in endodontics despite the increasing demand for CBCT examinations in dentistry [17]. It is noteworthy that in terms of diagnostic imaging of periapical abnormalities a profound difference is encountered when comparing dental and radiological practices. Dental practitioners more frequently use two-dimensional imaging techniques, namely PR and PAN, because of their high availability in dental offices. PR and PAN are well integrated into routine dental care because they are relatively simple to perform, cost-effective and provide adequate diagnostic information for many common dental conditions [18, 21]. Conversely, radiologists are more frequently engaged with PAN and CBCT examinations. In the last few years, CBCT has been reshaping imaging routines in endodontic field because CBCT is a volumetric technique offering higher spatial resolution—voxel size 0.075 mm to 0.4 mm—than traditional two-dimensional radiographs by revealing in full detail the extent of periapical bone lesions, root canal anatomy and potential root fractures [29, 52]. This enhanced visualization offers an extremely accurate representation of anatomical structures and facilitates more precise treatment planning and outcome assessment. Radiologists, equipped with CBCT units, can evaluate the extent and type of lesion with greater clarity and precision, aiding in the diagnosis of complex cases that might be missed or poorly defined by conventional radiographs [53, 54].

The difference in imaging practices between radiologists and dental practitioners is thus a reflection of the technological resources available, the specific diagnostic needs of each professional and the possibility of using two- or three-dimensional techniques in relation to what the law of each country states on the use of radiological devices in radiological clinics or dental private practices. While dental practitioners use PR and PAN for their practicality and effectiveness in general dental assessments, radiologists mostly use PAN and CBCT to achieve higher diagnostic accuracy in more complex or ambiguous cases [55, 56]. This divergence underscores the complementary role of dental and radiological practices in achieving comprehensive dental care because initial evaluations in dental offices can be supplemented by detailed radiological investigations when necessary. The preference for CBCT among radiologists also highlights the ongoing advancements in dental imaging technologies and their integration into clinical practice. Such volumetric technique not only enhances the detection and characterization of periapical bone lesions but also improves treatment outcomes by providing clinicians with precise information regarding the spatial relationships of anatomical features.

Nevertheless, the shift toward routine use of CBCT examinations raises concerns regarding radiation exposure, necessitating careful consideration of ALADA (As Low As Diagnostically Acceptable) principles [57]. As technology advances, strides are being made to optimize CBCT protocols in order to balance diagnostic benefits with radiation safety, potentially including the development of low-dose imaging techniques and machine learning algorithms for image analysis [31, 58]. However, the integration of CBCT examinations in endodontics should be limited to selected cases of AP lesions, specifically if complex treatments involving a surgical approach are needed [48].

The main limitation of the present review was that the accuracy values ​​were not always extracted directly from the text of the articles, but, in some cases, they were calculated using the data provided by the articles themselves. It could bring to the introduction of possible bias into the comparison between the different studies’ results. Nevertheless, the current study represented the first attempt to analyze the ability of PR and PAN to identify AP lesions by a systematic procedure.

## Conclusions

The analysis of the diagnostic accuracy of PR and PAN in the detection of AP lesions indicated that PR had an overall slightly higher diagnostic accuracy than PAN (71% vs 66%). This difference was mainly found in the upper/lower incisor areas and upper molar area where the diagnostic accuracy of PR and PAN was 67.0%–75.2% and 42.0%–70.0%, respectively. PR demonstrated better diagnostic accuracy in those anatomical areas in which PAN commonly has intrinsic limitations linked to the rotary image acquisition technology, which determine inevitable phenomena of overlapping and geometric distortion. Therefore, it is recommended to use PR in the diagnosis of AP lesions of the upper arch and lower incisor area, whereas the lower premolar and molar areas can be indiscriminately investigated with PR and PAN. In any case, two-dimensional imaging must be considered a first-level examination for the identification of AP lesions and planning of their treatment. Three-dimensional imaging techniques commonly used in dental practice as CBCT cannot replace PR and PAN for dosimetry reasons and should only be used in selected cases. Evidence from this review provided a useful tool to support radiologists and dentists in their decision-making when inflammatory periapical bone lesions are suspected in order to achieve the best clinical outcome for patients, improving the quality of clinical practice.

## Data Availability

The data presented in the study are publicly available in the literature.

## Appendix

Diagnostic accuracy, sensitivity, specificity, positive and negative predictive values of periapical radiography and panoramic radiography in the detection of apical periodontitis. Methods used to calculate them in the studies without a direct index. See Table 7.

**Table 7:** Diagnostic accuracy of PR and PAN in the detection of AP lesions and methods used to calculate them in the studies without a direct index

Study	Description of diagnostic accuracy measurement
Estrela et al., 2008 [50]	Diagnostic accuracy was 70.0% for PR and 54.0% for PAN. Sensitivity, specificity, PPV and NPV were 55.0%, 98.0%, 98.0% and 55.0% for PR and 28.0%, 100%, 99.0% and 44.0% for PAN, respectively
Estrela et al., 2009 [43]	In a sample of 1,020 analyzed teeth, AP lesions were detected in 397 teeth (38.9%) using PR and in 614 teeth (60.2%) using CBCT. The diagnostic accuracy was calculated with the ratio 397:614 = X:100, where "X" represented the diagnostic accuracy as a percentage of the PR compared to CBCT used as a reference standard. Diagnostic accuracy was 64.7% for PR
Moura et al., 2009 [44]	Percentage values ​​regarding AP lesions were provided in two different root canal obturation situations: 1–2 mm from the apex and 1–2 mm beyond the apex. The study analyzed three anatomical sites (anterior teeth, premolars and molars), using both PR and CBCT. To calculate diagnostic accuracy, averages were made between the percentages of the two-root canal obturation lengths for each anatomical area. This was done to avoid making distinctions between the two-root canal obturation lengths, as it was not relevant to the systematic review in question. From the calculated averages, it emerged that AP lesions were found on PR in 16.5%, 11.7% and 27.3% in the anterior, premolar and molar areas, respectively. In CBCT, after calculating the averages in the same way, the percentage values ​​were 24.5% in the anterior teeth, 14.5% in premolars and 36.3% in molars. Proportions were calculated to determine the diagnostic accuracy between PR and CBCT for each anatomical area, using CBCT as a reference standard. For example, for the anterior teeth, the ratio was 16.5: 24.5 = X:100, where "X" represents the diagnostic accuracy of PR. After calculating the three proportions, one for each anatomical area, it emerged that the diagnostic accuracy in anterior teeth was 67.3%, in premolars 80.3% and in molars 75.2%. Finally, the percentage accuracy values ​​were averaged to report an overall value. At the end, diagnostic accuracy was 74.3% for PR
Weissman et al., 2015[45]	It was reported that the probability of finding AP lesions was 56.7% with PR and 79.1% with CBCT. Since CBCT was considered the reference standard in this study, a proportion was made: 56.7:79.1 = X:100. Diagnostic accuracy was 71.7% for PR. To calculate the other parameters, AP lesions detected both by PR and CBCT were considered true positives (38) while those not detected on PR but visible on CBCT were considered false negatives (15). False positives were AP lesions detected on PR but not on CBCT (0) and true negatives when neither PR or CBCT detected any lesion (14). On this basis, sensitivity, specificity, PPV and NPV were 72.0%, 100%, 100% and 48.0%
Kanagasingam et al., 2017 [39]	Area under the curve (AUC) was provided as a measure of diagnostic accuracy for several imaging methods: FP (single intraoral film), FPS (3-projection intraoral film: normal, disto and mesio angled 10°), DP (single intraoral digital view), DPS (digital intraoral in the 3 projections). The diagnostic accuracy for the FP method was found to be 56.2%, for the FPS method 68.5%, for the DP method 62.9% and for the DPS method 68.8%. After averaging these values, an overall total diagnostic accuracy was 64.1% for PR using histopathological examination as the reference standard. Sensitivity, specificity, PPV and NPV were, again after averaging, 29.5%, 99.5%, 99.5% and 40.5%
Kanagasingam et al., 2017 [40]	Area under the curve was provided but only AP lesions in digital view and CBCT was considered. AUC value for DP method was 62.9%, for DPS method 68.8% and for CBCT 94.3%, using histopathological examination as a reference standard. An average was calculated between the two accuracy values ​​for the digital PR in the two methods. Diagnostic accuracy was 65.85% for PR. Sensitivity, specificity, PPV and NPV were reported for DP and DPS, and after averaging, the overall values were 32.5%, 99.0%, 99.0% and 41.5%
Nardi et al., 2017 [47]	Diagnostic accuracy was 65.0% for PAN. Sensitivity, specificity, PPV and NPV were 34.2%, 95.8%, 89.1% and 59.3%
Nardi et al., 2018 [48]	Diagnostic accuracy was 71.3% for PAN. Sensitivity, specificity, PPV and NPV were 48.8%, 93.8%, 88.6% and 64.7%
Nardi et al., 2020 [49]	Diagnostic accuracy values ​​of PAN in the detection of AP lesions were reported, both in treated and non-endodontically treated teeth. Diagnostic accuracy for endodontically treated teeth was 73.4%, while it was 67.6% for non-endodontically treated teeth. Average diagnostic accuracy was 70.0% for PAN. Likewise, average sensitivity, specificity, PPV and NPV were 45.9%, 96.3%, 92.4% and 64.2%
Jang et al., 2020 [46]	AP lesions were detected in 24.6% of cases using PR and in 35.5% of cases using CBCT. Since CBCT was considered the reference standard in this study, a proportion was calculated using CBCT as the reference, which was 24.6:35.5 = X:100. As result diagnostic accuracy was 69.4% for PR
Bosoni et al., 2021 [42]	Diagnostic accuracy was 72.1% for PAN. Sensitivity, specificity, PPV and NPV were 46.8%, 97.4%, 94.7% and 64.7%
Li et al., 2022 [41]	Data regarding the diagnosis of AP lesions using an AI-based method proposed in the same study were available. Using clinically diagnosed PR as a reference standard, the diagnostic accuracy of the AI ​​method was calculated. By adding the true positives and true negatives and dividing the result by the total number of cases—true positives and true negatives and false positives and false negatives—diagnostic accuracy was 83.0% for PR by AI. Sensitivity, specificity, PPV and NPV were 82.0%, 84.0%, 83.7% and 82.4%
